# *Enterococcus faecalis* FK-23 affects alveolar-capillary permeability to attenuate leukocyte influx in lung after influenza virus infection

**DOI:** 10.1186/2193-1801-2-269

**Published:** 2013-06-20

**Authors:** Kazutake Fukada, Daisuke Fujikura, Yosuke Nakayama, Masatoshi Kondoh, Takashi Shimada, Tadaaki Miyazaki

**Affiliations:** Division of Bioresources, Research Center for Zoonosis Control, Hokkaido University, Sapporo, 001-0020 Japan; Central Research Laboratories, Nichinichi Pharmaceutical Corporation Ltd., 239-1 Tominaga, Iga, Mie, 518-1417 Japan; Department of Probiotics Immunology, Institute for Genetic Medicine, Hokkaido University, Sapporo, 060-0815 Japan; Division of Infection and Immunity, Research Center for Zoonosis Control, Hokkaido University, Sapporo, 001-0020 Japan

**Keywords:** H1N1 influenza virus infection, Lactic acid bacteria, *Enterococcus faecalis*, Alveolar-capillary permeability, Leukocyte influx

## Abstract

Infection with influenza A virus, one of the most common life-threatening viruses, causes the accumulation of inflammatory cells in the lung, which is directly correlated with influenza-associated morbidity and mortality. In this study, we investigated the potential of lysozyme-treated *Enterococcus faecalis* FK-23 (LFK) to prevent influenza in influenza virus-infected mice. C57BL/6N mice were orally administered LFK and intranasally infected with influenza virus A/Puerto Rico/8/34 (H1N1) at lethal doses. After infection with influenza A virus, the survival rate of the LFK-administered mice was significantly higher than that of saline-administered mice. Staining of lung sections with hematoxylin-eosin, and cell counts of lung and bronchoalveolar lavage fluid showed that oral administration of LFK suppressed the excessive infiltration of leukocytes into the lung after viral infection. Extravasation assay revealed that the arrest was mediated by modulation of pulmonary alveolar-capillary permeability. Expression levels of genes involved in matrix degradation, which are correlated with vascular permeability, were downregulated in LFK-administered mice. These findings suggest that stabilizing the integrity of the alveolar-capillary barrier by the administration of LFK improves survival rate.

**Electronic supplementary material:**

The online version of this article (doi:10.1186/2193-1801-2-269) contains supplementary material, which is available to authorized users.

## Background

Influenza is a highly contagious acute respiratory disease caused by influenza viruses, which belong to the *Orthomyxoviridae* family. According to reports issued by the World Health Organization, approximately 5–15% of the world’s population is annually infected with influenza A virus, and 250, 000–500, 000 of these infected patients die each year. Annual immunization is the primary means to protect from influenza virus infection, but this vaccination strategy can be limited by the production time ( Boltz et al. 2010 ). In addition to the vaccination strategy, antiviral therapy is useful to control the spread of influenza. Two classes of antiviral drugs (M2-ion channel inhibitors and neuraminidase inhibitors) have been approved for the prevention and treatment of influenza ( Boltz et al. 2010 ; van der Vries et al. 2011 ). However, the effectiveness of these antiviral agents may be limited by the rapid emergence of drug-resistant viruses ( van der Vries et al. 2011 ).

Severe influenza virus infection can lead to diffuse alveolar damage, which is characterized by pulmonary edema and the accumulation of inflammatory cells in the lung, with histopathologic features of acute lung injury (ALI) and acute respiratory distress syndrome (ARDS), the most severe form of ALI. These lung injuries directly correlate with influenza-associated morbidity and mortality because of the impairment of gas exchange and respiratory functions. ARDS is characterized by an increase in the permeability of the alveolar-capillary barrier, which is formed by the microvascular endothelium and the alveolar epithelium, leading to an influx of fluid and leukocytes into the alveolar airspace across both the endothelium and the epithelium ( Nunes 2005 ). ALI and ARDS were leading causes of death following infections with pandemic 2009 H1N1 and highly pathogenic avian H5N1 influenza viruses ( Perrone et al. 2008 ; Zhang et al. 2012b ).

Several experimental studies have shown that influenza-induced death is suppressed by anti-inflammatory agents that counteract the inflammatory response of the hosts without affecting virus replication itself ( Darwish et al. 2011 ; Garcia et al. 2010 ). These reports indicate that suppression of the heightened inflammatory response to viral infection is important in order to avoid influenza-caused death.

Oral or intranasal administrations of lactic acid bacteria are effective against influenza A virus infection ( Izumo et al. 2010 ; Maeda et al. 2009 ). These bacterial administrations help to enhance host’s immune response that causes the reduction of the viral replication efficiency and/or upregulation of cytokine expression. Previously, we reported that the water-soluble fraction of lysozyme-treated lactic acid bacterium *Enterococcus faecalis* FK-23 (LFK) reduces the mortality associated with influenza A virus infections ( Kondoh et al. 2012 ). However, the mechanism underlying the anti-influenza effect of LFK remains unclear. We previously reported that the oral administration of LFK attenuates the eosinophil influx into the upper airway in a murine allergic model ( Zhu et al. 2012 ) and the inflammatory cell influx into bronchoalveolar lavage fluid (BALF) in a murine asthmatic model ( Zhang et al. 2012a ). These results implied the possibility of anti-inflammatory effect of LFK during influenza virus infection. In this study, we demonstrate that the administration of LFK reduces mortality after H1N1 viral infection and suppresses the excessive influx of leukocytes, which cause inflammatory reactions, into lungs via modulation of the alveolar-capillary permeability.

## Results

### Reduction of the mortality of influenza virus-infected mice by LFK administration

**Figure 1:**
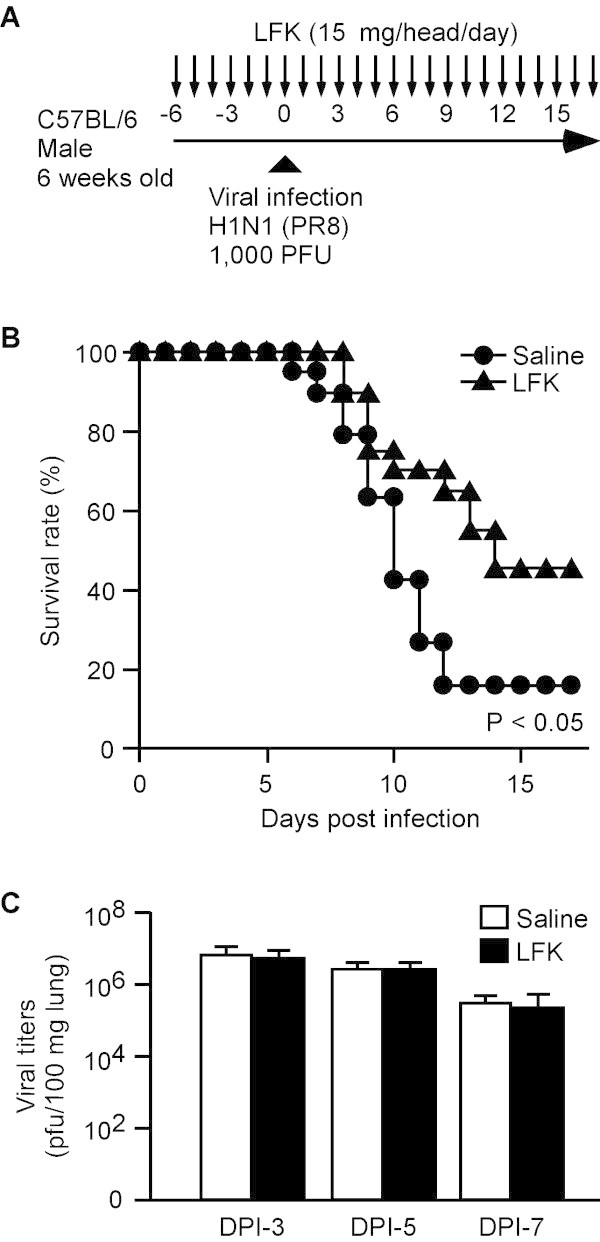
**Administration of LFK protects mice from influenza virus PR8-induced death.** (**A**) The experimental protocol for oral administration and infection with influenza A/PR/8. The vertical arrows indicate oral administration of LFK (15 mg/head, dissolved in 200 μl saline) or saline from day -6 to day 17. The triangle on day 0 indicates the inoculation with PR8 (1,000 PFU/mouse). (**B**) Survival rate of mice after the infection with PR8 (saline-administered group: circle, *n* = 19; LFK-administered group: triangle, *n* = 20). Statistically significant difference between saline and control mice is indicated by P value (P < 0.05 by the log-rank test). (**C**) Viral titers in the lungs after inoculation with PR8. Mean viral titers ± standard deviations are shown. Each group consists of 6 to 7 mice.

To test for preventive effect against influenza, we orally administered LFK to mice at a dose of 15 mg per mouse, once daily, for 6 days before and 17 days after the viral infection, and monitored the survival rate for 17 days after the infection (Figure 1A). In this experiment, the LFK dose was fixed at 15 mg per mouse as previous report in which water-soluble fraction of LFK suspension (15 mg per mouse) was administered to mice ( Kondoh et al. 2012 ). However, in this experiment, LFK suspension rather than water-soluble fraction of LFK was administered for comprehensive understanding of the mechanism for prevention of influenza by LFK. As shown in Figure 1B, 16% of mice in the control group, which were administered saline orally, survived for 17 days post-infection. In contrast, 45% of mice administered LFK orally survived after infection. This result indicates that oral administration of LFK provides effective protection from lethal infection by influenza A virus.

Next, we examined whether the improved survival was due to a reduction in the viral replication efficiency in the lung, because oral or intranasal administration of lactic acid bacteria was shown to suppress viral replication efficiency in the lung ( Maeda et al. 2009 ; Izumo et al. 2010 ). In contrast to these reports, the viral titer in lungs showed no significant difference between the saline- and LFK-administered groups at 3 days, 5 days, and 7 days post-infection (DPI) (Figure 1C). This result suggests that the mechanism by which oral administration of LFK protects against virus-induced death is different from that reported for other lactic acid bacteria.

### Suppression of the infiltration of leukocytes into lung after virus-infection by LFK treatment

**Figure 2:**
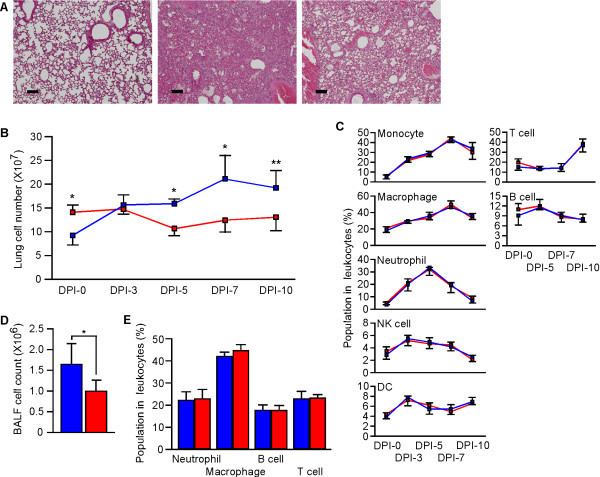
**Administration of LFK suppresses the leukocytes infiltration into the lungs after PR8 viral infection.** (**A**) Histology of lung tissue section stained with HE at DPI-7 (left: non-infected control, middle: saline-administered mice at DPI-7, right: LFK-administered mice at DPI-7). Original magnification is X10. Scale bars indicate 100 μm. (**B**) The lung cells were isolated at DPI-0, DPI-3, DPI-5, DPI-7 and DPI-10, and the absolute number of lung cells was counted (*: P < 0.01, **: P < 0.05, Student’s *t* test). Blue and red squares indicate saline- and LFK-administered groups, respectively (DPI-0: saline *n* = 7, LFK *n* = 7; DPI-3: saline *n* = 5, LFK *n* = 4; DPI-5: saline *n* = 5, LFK *n* = 4; DPI-7: saline *n* = 5, LFK *n* = 5; DPI-10: saline *n* = 3, LFK *n* = 5). (**C**) The change in lung cell population (blue: saline-administered group, red: LFK-administered group) was analyzed through a flow cytometer (DPI-0: saline *n* = 7, LFK *n* = 7; DPI-3: saline *n* = 5, LFK *n* = 4; DPI-5: saline *n* = 5, LFK *n* = 4; DPI-7: saline *n* = 5, LFK *n* = 5; DPI-10: saline *n* = 3, LFK *n* = 5). (**D**) The total number of BALF cells at DPI-5 was counted (blue: saline-administered group, *n* = 8; red: LFK-administered group, *n* = 6; *, P < 0.05, Student’s *t* test). (**E**) Cell population in BALF was analyzed through a flow cytometer (blue: saline-administered group, *n* = 8; red: LFK-administered group, *n* = 6).

Leukocytes, including inflammatory cells, mononuclear cells, and lymphocytes, infiltrate into the lung area after viral infection ( Kohlmeier and Woodland 2009 ; Fukushi et al. 2011 ). To gain insight into the protective mechanism underlying LFK activity, we stained the lung sections using hematoxylin-eosin (HE). This staining revealed that infiltration of leukocytes into the pulmonary parenchyma was suppressed at DPI-7 in the LFK-administered group, whereas infiltration of leukocytes in the pulmonary parenchyma and alveolar collapse were observed in the saline-administered group (Figure 2A and Additional file 1: Figure S1). To confirm that LFK suppresses the infiltration of leukocytes, we homogenized the removed lungs and counted the number of whole lung cells by using a microscope. In the saline-administered group, the number of lung cells increased after viral infection and reached a maximum at DPI-7 (Figure 2B). Unlike in the control group, the number of these cells in the LFK-administered group was significantly suppressed at DPI-5, DPI-7, and DPI-10 (Figure 2B). Similar results were obtained for BALF cells. The total cell number in the BALF was suppressed in the LFK-administered group at DPI-5 compared to that in the saline-administered group (Figure 2D).

### Upregulation of type II pneumocyte number by LFK administration

Surprisingly, the number of whole lung cells at DPI-0 was significantly higher in mice that were administered with LFK for 6 days before viral infection than in the saline-administered group (Figure 2B). The alveolar septa consists of 2 types of pneumocytes (type I and II), connective tissues, and blood vessels ( Rogers 2010 ). We performed an immunohistochemical analysis because, on the basis of our observation of the HE-stained lung sections at DPI-0 (Additional file 2: Figure S2A), we expected an increase in the number of type II pneumocytes. Staining with type II pneumocyte marker (proSP-C, prosurfactant protein-C) showed that the number of type II pneumocyte was upregulated in the LFK-administered group compared to that in the saline-administered group (Additional file 2: Figures S2B and S2C).

### Suppression of cellular infiltration independent of cell type

Next, we evaluated which types of cells infiltrate into the lung area after viral infection. Cell populations of each leukocyte in the lung were analyzed at DPI-0, DPI-3, DPI-5, DPI-7, and DPI-10 using a flow cytometer. In the early phase of infection with influenza virus, innate immune cells, such as monocytes, macrophages, and neutrophils, were recruited to the lung at DPI-5 and DPI-7 (Figure 2C). Thereafter, T cell migration was observed (Figure 2C). However, there was no significant difference between the saline- and LFK-administered groups in the population of the infiltrating cells. A similar result was obtained for the cell population in the BALF at DPI-5. The saline- and LFK-administered groups showed no significant difference in the population of leukocytes that infiltrated the alveolar space (Figure 2E). These results indicate that the suppression of cellular infiltration into the lung occurred in all types of leukocytes that we analyzed.

### Gene expression of chemokines and cytokines after virus infection

**Figure 3:**
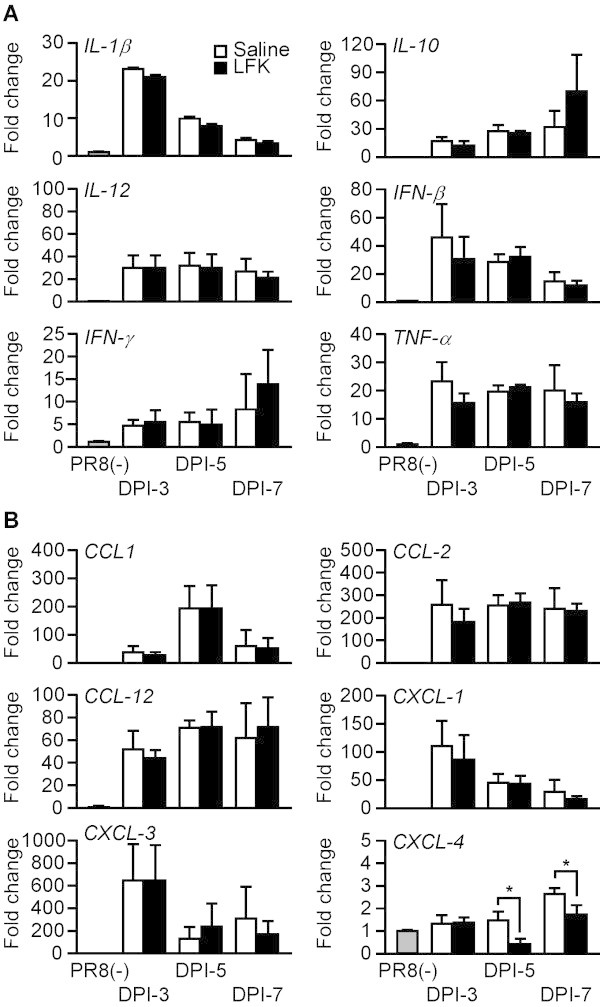
**Cytokine and chemokine mRNA expression levels during the course of the viral infection.** (**A**) Cytokine mRNA expression level in lung. (**B**) Chemokine mRNA expression level in lung. Each value is expressed as fold change compared to non-infected control mice (*: P < 0.01, Student’s *t* test). Gray, white, and black columns indicate non-infected control (*n* = 3), saline-administered (*n* = 6), and LFK-administered (*n* = 6) groups, respectively.

Chemokines regulate the trafficking of various types of leukocytes, and cytokines can induce the production of chemokines ( Kohlmeier and Woodland 2009 ). Therefore, we next evaluated the mRNA expression level of cytokines and chemokines in the lung during the course of viral infection. The expression level of various Th1 cytokines, pro-inflammatory cytokines, CCL-chemokine ligands, and CXCL-chemokine ligands were elevated after the viral infection (Figure 3A, 3B, and Additional file 3: Figure S3A and S3B). However, no significant difference in the expression level of cytokines and chemokines was observed except for CXCL4 (platelet factor 4), which on its own does not show any chemotactic activity to leukocytes ( Kasper and Petersen 2011 ).

### Suppression of virus-induced alveolar-capillary permeability by LFK treatment

**Figure 4:**
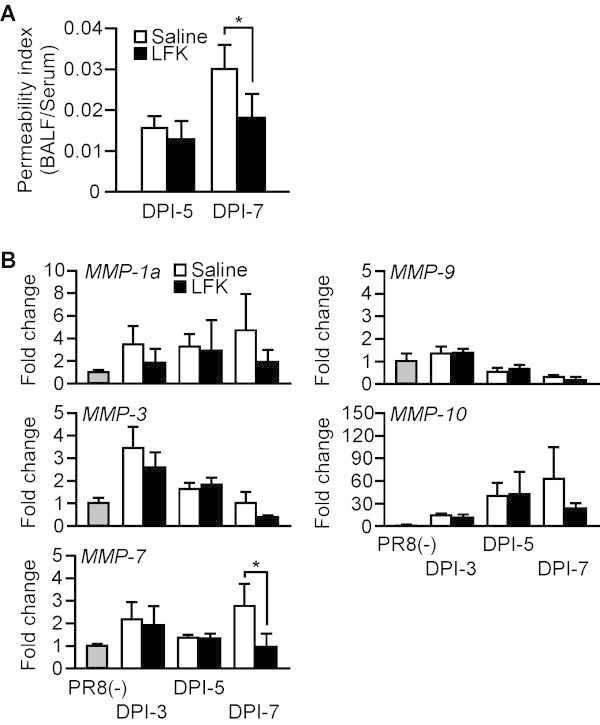
**Administration of LFK suppresses the virus-induced alveolar-capillary permeability in lungs.** (**A**) Alveolar-capillary permeability was assessed by extravasation of Evans blue dye into BALF at DPI-5 and DPI-7. Permeability index was determined from the dye content of BALF compared with serum (DPI-5: saline *n* = 5, LFK *n* = 6; DPI-7: saline *n* = 5, LFK *n* = 5; *: P < 0.05, Student’s *t* test). (**B**) MMP mRNA expression level in lung. Each value is expressed as fold change compared to non-infected control mice (*: P < 0.05, Student’s *t* test). Gray, white, and black columns indicate non-infected control (*n* = 4), saline-administered (*n* = 4), and LFK-administered (*n* = 4) groups, respectively.

Next, we examined whether the suppression of leukocyte migration was due to the changes in pulmonary alveolar-capillary permeability, because pulmonary inflammation during viral infection is closely correlated with the breakdown of pulmonary barrier integrity ( Steinberg et al. 2011 ; Fukushi et al. 2011 ). The change in alveolar-capillary permeability was assessed by monitoring the extravasation of Evans blue dye into the BALF. As shown in Figure 4A, increases in virus-induced Evans blue accumulation were suppressed at DPI-7 in the LFK-administered mice. This indicates that the administration of LFK suppresses viral infection-induced pulmonary permeability, and that the suppression of inflammatory cell influx by LFK is due to stabilization of the alveolar-capillary barrier integrity.

### Modulation of gene expression controlling the vascular permeability by LFK administration

Mechanisms that control vascular permeability during inflammation have been reported. Matrix metalloproteinases (MMPs) are important regulatory enzymes in pro-inflammatory pathways, and their expression and activities are typically increased during the inflammatory process. MMPs degrade major components of the vascular basement membrane and extracellular matrix, such as gelatin and collagen, in the alveolar-capillary barrier ( Manicone and McGuire 2008 ). Therefore, we investigated the mRNA expression level of MMPs in the lung during the course of viral infection. MMP-7 expression following viral infection was suppressed in the LFK-administered group at DPI-7 (Figure 4B). For the other MMPs, a decreasing trend was observed in the LFK-treated group at DPI-7 (Figure 4B). These results suggest that the downregulation of MMP expression by LFK-administration could attenuate the vascular permeability caused by viral infection.

## Discussions

A number of probiotic bacteria, including *Bifidobacteria* and lactic acid bacteria such as *Lactobacillus*, have an anti-influenza effect that is achieved by the reduction of viral replication efficiency and/or upregulation of Th1 cytokine expression through the activation of the host’s immune system ( Harata et al. 2010 ; Yasui et al. 2004 ; Maeda et al. 2009 ; Izumo et al. 2010 ). We previously reported that oral administration of the soluble components of LFK, lysozyme-treated lactic acid bacteria *E. faecalis* FK-23, has an anti-influenza effect by upregulation of the anti-inflammatory cytokine IL-10 in lung ( Kondoh et al. 2012 ). However, administration of the LFK suspension did not markedly upregulate IL-10 expression in this study (Figure 3A). This discrepancy could be due to the differences in composition between administered samples. In this report, LFK suspension was administered to mice, while the supernatant of LFK suspension after centrifuge was used in the previous report ( Kondoh et al. 2012 ). The present study demonstrated that LFK suspension has the ability to neither repress the viral replication, nor enhance the immune system in the lungs (Figures 1C, 3, and Additional file 3: Figure S3). These results indicate that the mechanism by which oral administration of LFK protects against virus-induced death is different from that reported for other probiotic bacteria.

Pneumocytes play pivotal roles in pulmonary function: (1) lung surfactants secreted by pneumocytes prevent alveolar collapse ( Griese 1999 ), (2) type II pneumocytes are progenitor cells for alveolar epithelium during injuries ( Mason 2006 ), and (3) type I pneumocytes are responsible for oxygen and carbon dioxide exchange ( Mason 2006 ). In Figure S2, we demonstrated that oral administration of LFK increases the number of type II pneumocytes, which act as alveolar epithelial stem cells ( Fehrenbach 2001 ). However, further experiments, such as immunohistochemical analysis using antibodies against proliferation marker and mitogen, are required to clear how the proliferation is induced by LFK. Influenza virus infection against type II pneumocyte causes damage to the alveolar epithelial cells and/or the cell death ( Matute-Bello et al. 2001 ; van Riel et al. 2007 ; Loosli et al. 1975 ). The damage and/or loss result in disturbances in fluidity at the alveolar-capillary border, which is associated with gas exchange inefficiency and pulmonary vascular permeability ( Mura et al. 2010 ; Ware and Matthay 2000 ; Matute-Bello et al. 2001 ). Therefore, the increase in the number of type II pneumocytes in the LFK-administered group suggests that LFK might have the effect of improving respiratory function and epithelial barrier function by the proliferation and/or differentiation of type II pneumocyte during viral infection. Another possibility is that the increase in the number of type II pneumocytes might cause the lower multiplicity of the virus in the virus-infected pneumocyte, which leads to the repression of cell death of type II pneumocyte and successive vascular permeability. However, further analysis of the induction of cell death, proliferation, and differentiation in the type II pneumocyte is required to unmask the role of the increased pneumocyte in LFK-administered mice during the influenza virus infection.

Cytokines and successive chemokines are produced following influenza virus infection in the lungs as early anti-viral responses ( Kohlmeier and Woodland 2009 ). In the present study, the expression of cytokines and chemokines after viral infection were not modulated by the treatment with LFK, except for CXCL4 (Figure 3), although the reported probiotic bacteria regulate the expressions as a viral defense mechanism ( Maeda et al. 2009 ; Yasui et al. 2004 ). This is supported by our unpublished data that show that elimination of peptidoglycan from the cell of *E. faecalis* FK-23 by enzymatic degradation results in loss of the ability to produce various cytokines in ex vivo culture of the splenocytes from mice (data not shown). Increased influx of leukocytes into alveolar space occurs after viral infection, and leukocyte extravasation from the blood to the inflamed tissue is a controlled process involving rolling, adhesion, and migration ( Weber et al. 2007 ). While CXCL4 shows no chemotactic activity against leukocytes, CXCL4 enhances monocyte adhesion to the endothelium and neutrophil extravasation to the inflamed lung via heterophilic interaction with CCL5 ( Grommes et al. 2012 ; von Hundelshausen et al. 2005 ; Kasper and Petersen 2011 ). Therefore, our results suggest that the suppression of leukocyte migration into the lung by the administration of LFK is partially mediated by the downregulation of CXCL4 expression.

MMPs induced by influenza virus infection degrade the components of the vascular basement membrane and extracellular matrices, and the resulting dysfunction of the alveolar-capillary barrier contributes to pathogenesis ( Ng et al. 2012 ; Wang et al. 2010 ; Davey et al. 2011 ; Manicone and McGuire 2008 ). MMP-7 is reported to degrade not only VE-cadherin but also E-cadherin, which are major components of endothelial and epithelial adherens junctions, respectively ( McGuire et al. 2003 ; Ichikawa et al. 2006 ). These adherens junctions act as gatekeepers for the passage of leukocytes during inflammation. Furthermore, stabilization of vascular barrier integrity by upregulating the expression of the VE-cadherin mRNA reduces mortality after H5N1 viral infection and suppresses leukocyte influx into the lung ( London et al. 2010 ). Therefore, our results suggest that downregulation of the expressions of MMP-7 and other MMPs by LFK attenuates not only the breakdown of adherens junctions of alveolar epithelial and vascular endothelial cells, but also degradation of the vascular basement membrane and extracellular matrices. However, further analyses for protein level of MMP and MMP-mediated degradation of target proteins would be required for comprehensive understanding of the effect of LFK for the prevention of influenza.

The components of LFK to exert the anti-influenza effect remain unidentified. Since a recent study has demonstrated that the soluble peptidoglycan of commensal bacteria translocates from the intestine to the systemic circulation ( Clarke et al. 2010 ), one possibility is that the lysozyme-degraded components of peptidoglycan could translocate to the lungs, thereby modulating the alveolar-capillary barrier. Identification of the active components in LFK will be essential to further understand the precise anti-influenza mechanism.

In this study, we demonstrated that the administration of LFK improved survival rates and suppressed the infiltration of leukocytes into the lung after viral infection. Several factors could contribute to the effect of LFK against influenza virus infection. These include the suppression of leukocyte infiltration into the lungs by both the downregulation of CXCL4 expression and the modulation of pulmonary endothelial-epithelial permeability via the increase of type II pneumocytes and the repression of MMPs expression. Our findings support the notion that suppression of the breakdown of the alveolar-capillary barrier and the subsequent leukocyte influx into the lung would improve the survival rate after viral infection. Stabilization of pulmonary alveolar-capillary barrier integrity using bacterial components might be a useful strategy for managing seasonal and pandemic influenza.

## Methods

### LFK preparation

*E. faecalis* strain FK-23 was cultured in broth medium containing 2.5% of glucose, 1.4% of yeast extract, 0.8% of peptone, and 4.4% of K_2_HPO_4_ for 18h at 37°C, and the cultures were harvested by centrifugation. After washing with distilled water, the bacteria were treated with lysozyme, and then the reaction mixture was heated to 110°C for 10 min before lyophilization as described previously ( Kondoh et al. 2012 ). LFK (15 mg/head, dissolved in 200 μl saline) or saline (200 μl) was orally administered using a feeding needle once a day.

### Virus preparation

An influenza virus strain, A/Puerto Rico/8/34 (H1N1; PR8) was used in this study. The infectious materials were handled in a biosafety level 2 facility under approved protocols in accordance with guidelines of Hokkaido University. The virus was prepared as described previously ( Kondoh et al. 2012 ). In brief, the virus was propagated in the allantoic cavities of 10-day-old embryonated chicken eggs at 35°C for 48 h, and then was concentrated and purified by density gradient centrifugation. The purified virus was suspended in phosphate-buffered saline (PBS) and stored at -80°C until use.

### Mice and viral infection

Male C57BL/6N mice (6 weeks old) were purchased from CLEA Japan. Mice were housed in isolator cages in a biosafety level 2 room (12 h light/dark cycle) with free access to standard diet (CE-2; CLEA Japan) and tap water. We performed animal care and experiments in accordance with guidelines and approval of the Animal Care and Use Committee of Hokkaido University (08–0231). All surgery was performed under isoflurane anaesthesia. Mice were lightly anesthetized with isoflurane (Dainippon Pharmaceutical, Osaka, Japan) and inoculated intranasally with 10^3^ PFU at 50 μl in both nostrils on day 0. The survival rate and body weight were monitored daily until 17 days after the viral infection. To minimize suffering after viral challenge, mice were carefully observed each day and mice reaching approved endpoint criteria were euthanized by overdose of isoflurane. Mice were euthanized by overdose of isoflurane on day 17 after the infection.

### Viral titration

Plaque forming assay was performed as described previously ( Fukushi et al. 2011 ) with some modifications of the protocol. Mice were sacrificed by isoflurane inhalation and lung was removed from the mice. The lungs were completely homogenized in 1X MEM medium using Micro smash (Tomy Seiko, Tokyo, Japan), and the homogenates were serially diluted with cold PBS. For a plaque assay, Madin-darby canine kidney (MDCK) cells were plated in a flat bottomed 12 well plate 24 h before infection. Supernatants from lung homogenates serially diluted were used to infect the confluent MDCK cells at 37°C for 1h. The cells were washed and subsequently overlaid with MEM mixed with 0.8% Bacto-agar (Difco, Sparks, USA) in the presence of trypsin (5 μg/ml). The plates were incubated at 35°C for 2 days and the plaques were counted.

### Histological analysis

Mice were sacrificed by isoflurane inhalation and lung was removed from the mice. Removed lungs were immediately fixed in 10% PBS-buffered formalin. Paraffin-embedded tissues were sectioned to a thickness of 4 μm and stained with hematoxyline and eosin (Merck, USA) using standard histological techniques. For immunehistochemical study, the paraffin-embedded tissue sections were incubated with anti-prosurfactant protein C antibody which is a type II pneumocyte marker (Millipore, Billerica, USA), incubated with horseradish peroxidase labeled polymer anti rabbit system (DAKO, Glostrup, Denmark), developed with envision + system HRP labeled polymer anti-rabbit (DAKO Glostrup, Denmark), and counterstained with hematoxylin (Merck, USA) in accordance to manufacturer’s instructions. The proportion of prosurfactant protein C positive cells was calculated by counting the number of hematoxyline stained cells and its positive cells in 6 random microscopic fields at a magnification of 40.

### Flow cytometric analysis of lung and BALF cells

Mice were sacrificed by isoflurane inhalation and lung was removed from the mice. Lung tissue was homogenized using gentleMACS dissociator (Miltenyi Biotech, Bergisch Gladbach, Germany) in C tube (Miltenyi Biotech, Bergisch Gladbach, Germany) containing HEPES buffer (pH 7.4) with 2 μg/ml of collagenase-D (Roche, Basel, Switzerland) and 40 U/ml of DNase I (Takara bio, Otsu, Japan), and then incubated for 30 min at 37°C with gentle rotation. After centrifugation, cells were treated with lysis buffer (BD Biosciences, Franklin lakes, USA) according to manufacture’s instruction. The bronchoalveolar lavage fluid (BALF) cells were collected using a 24G catheter (Terumo, Tokyo, Japan) by insertion of PBS from the trachea. Number of lung and BALF cells was counted using a Neubauer hemocytometer (Erma, Tokyo, Japan). Cells were incubated with monoclonal antibody 2.4G2, and stained with FITC-conjugated anti-CD3e (BD Biosciences, clone 145-2C11) for T-cell (CD3e^+^) analysis, with FITC-CD11b (BD Biosciences, clone MI/70) and PE-Ly6G (BD Biosciences, clone 1A8) for neutrophils (CD11b^high^ Ly6G^high^) and monocytes (CD11b^high^ Ly6G^med^) analyses, with FITC-CD49b (BD Biosciences, clone DX5) for NK cells (CD49b^+^) analysis, with FITC-B220 (Biolegend, clone RA3-6B2) for B cells (B220^+^) analysis, or with APC-CD11c (Biolegend, clone N418) and PE/Cy7-F4/80 (Biolegend, clone BM8) for dendritic cell (CD11c^+^ F4/80^-^) and macrophages (F4/80^+^) analyses. The stained cells were analyzed with FACSCanto II flow cytometer (BD Biosciences).

### Real time PCR

Real time PCR reaction was performed as described previously ( Kondoh et al. 2012 ). In brief, total RNA was extracted from whole lung homogenates using TRIzol reagent (Invitrogen, Carlsbad, USA) and Micro smash (Tomy Seiko, Tokyo, Japan), and then treated with DNase I (Takara bio, Otsu, Japan). The cDNA was synthesized using oligo dT20 primers (Toyobo, Osaka, Japan), random primers (Toyobo, Osaka, Japan) and ReverTra Ace (Toyobo, Osaka, Japan). Each procedure was performed according to the manufacture’s instructions. Real time PCR was performed using SYBR Premix Ex Taq II (Takara bio, Otsu, Japan) with the MX3000P real time PCR system (Stratagene, La Jolla, USA). Cycling conditions were used as: 95°C for 10 sec to activate DNA polymerase, followed by 40 cycles of 95°C for 5 sec and 60°C for 30 sec. Expression levels of each mRNA were represented as relative expression amounts, which were normalized with glyceraldehyde-3-phosphate dehydrogenase (GAPDH).

### Measurement of alveolar-capillary permeability

Alveolar-capillary leakage after viral infection was determined using Evans blue dye as described previously ( Rhein et al. 2008 ). Two hours before killing, mice were injected intravenously via retro orbital sinus under isoflurane anaesthesia with 0.2 ml of 5 mg/ml Evans blue in PBS. BALF and serum were collected after killing mice with isoflurane and the optical density was determined at 600 nm. The permeability changes were evaluated by the BALF/serum concentration ratio.

### Statistical analysis

The data are expressed as means ± standard deviations (SD). A student’s *t* test was used for statistical analysis. The Kaplan-Meier method with the log-rank test was used for analysis of mortality. P value of <0.05 was considered to be significant.

## Electronic supplementary material

Additional file 1: Figure S1: Histology of lung tissue section stained with HE at DPI-7. Original magnification is X20 (A: non-infected control, B: saline-administered mice at DPI-7, C: LFK-administered mice at DPI-7). Scale bars indicate 100 μm. In each group, two fields, which were taken from lung section of different mice, are shown. (PDF 735 KB)

Additional file 2: Figure S2: Increased number of Type II pneumocyte by the administration of LFK. (A) Histology of lung tissue section stained with HE at DPI-0 (top: saline-administered mice, bottom: LFK-administered mice). Original magnification is X4. Scale bars indicate 250 μm. (B) Immunohistochemical staining of lung tissue section at DPI-0 using prosurfactant protein C (proSP-C) antibody (top: saline-administered mice, bottom: LFK-administered mice). Arrowheads indicate examples of proSP-C-positive cells. Original magnification is X40. Scale bars indicate 50 μm. (C) The proportion of proSP-C positive cells was counted in 6 random microscopic fields for each group at a magnification of X40 (blue: saline, red: LFK; *: P < 0.01, Student’s *t* test). (PDF 398 KB)

Additional file 3: Figure S3: Cytokine and chemokine mRNA expression levels during the course of the viral infection. (A) Cytokine mRNA expression level in lung. (B) Chemokine mRNA expression level in lung. Each value is expressed as fold change compared to non-infected control mice (*: P < 0.01, Student’s *t* test). Black, blue, and red columns indicate non-infected control (*n* = 3), saline-administered (*n* = 6), and LFK-administered (*n* = 6) groups, respectively. (PDF 85 KB)

## Abbreviations

ALI: Acute lung injury
ARDS: Acute respiratory distress syndrome
BALF: Bronchoalveolar lavage fluid
DPI: Days post-infection
HE: Hematoxylin-eosin
LFK: Lysozyme-treated *Enterococcus faecalis* FK-23
MDCK: Madin-darby canine kidney
MMP: Matrix metalloproteinase
SP-C: Prosurfactant protein-C.
