# Supplementary material for: Enterococcus faecalis FK-23 affects alveolar-capillary permeability to attenuate leukocyte influx in lung after influenza virus infection
Source: Springerplus. 2013 Jun 20;2:269. doi: 10.1186/2193-1801-2-269 (PMC3698428; doi:10.1186/2193-1801-2-269)
Supplement: Supplementary file 1 — Additional file 1: Figure S1: Histology of lung tissue section stained with HE at DPI-7. Original magnification is X20 (A: non-infected control, B: saline-administered mice at DPI-7, C: LFK-administered mice at DPI-7). Scale bars indicate 100 μm. In each group, two fields, which were taken from lung section of different mice, are shown. (PDF 735 KB) [file 40064_2013_347_MOESM1_ESM.pdf]

Additional file 1: Figure S1

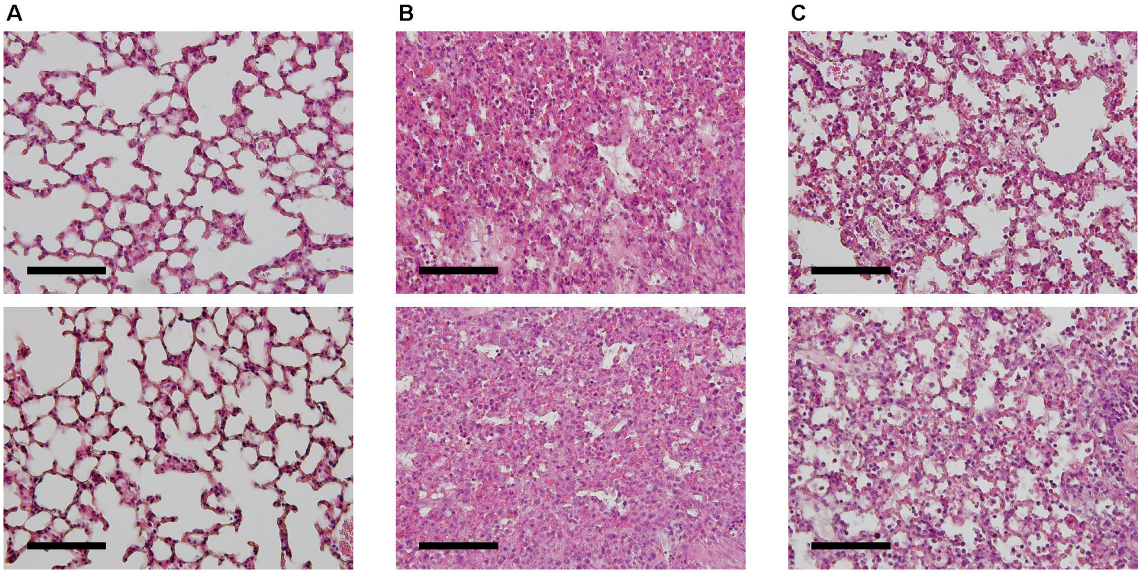

Figure S1 Histology of lung tissue section stained with HE at DPI-7.
Original magnification is X20 (A: non-infected control, B: saline-administered mice at DPI-7, C: LFK-administered mice at DPI-7). Scale bars indicate 100 μm. In each group, two fields, which were taken from lung section of different mice, are shown.
