# Supplementary material for: Enterococcus faecalis FK-23 affects alveolar-capillary permeability to attenuate leukocyte influx in lung after influenza virus infection
Source: Springerplus. 2013 Jun 20;2:269. doi: 10.1186/2193-1801-2-269 (PMC3698428; doi:10.1186/2193-1801-2-269)
Supplement: Supplementary file 2 — Additional file 2: Figure S2: Increased number of Type II pneumocyte by the administration of LFK. (A) Histology of lung tissue section stained with HE at DPI-0 (top: saline-administered mice, bottom: LFK-administered mice). Original magnification is X4. Scale bars indicate 250 μm. (B) Immunohistochemical staining of lung tissue section at DPI-0 using prosurfactant protein C (proSP-C) antibody (top: saline-administered mice, bottom: LFK-administered mice). Arrowheads indicate examples of proSP-C-positive cells. Original magnification is X40. Scale bars indicate 50 μm. (C) The proportion of proSP-C positive cells was counted in 6 random microscopic fields for each group at a magnification of X40 (blue: saline, red: LFK; *: P < 0.01, Student’s t test). (PDF 398 KB) [file 40064_2013_347_MOESM2_ESM.pdf]

Additional file 2: Figure S2

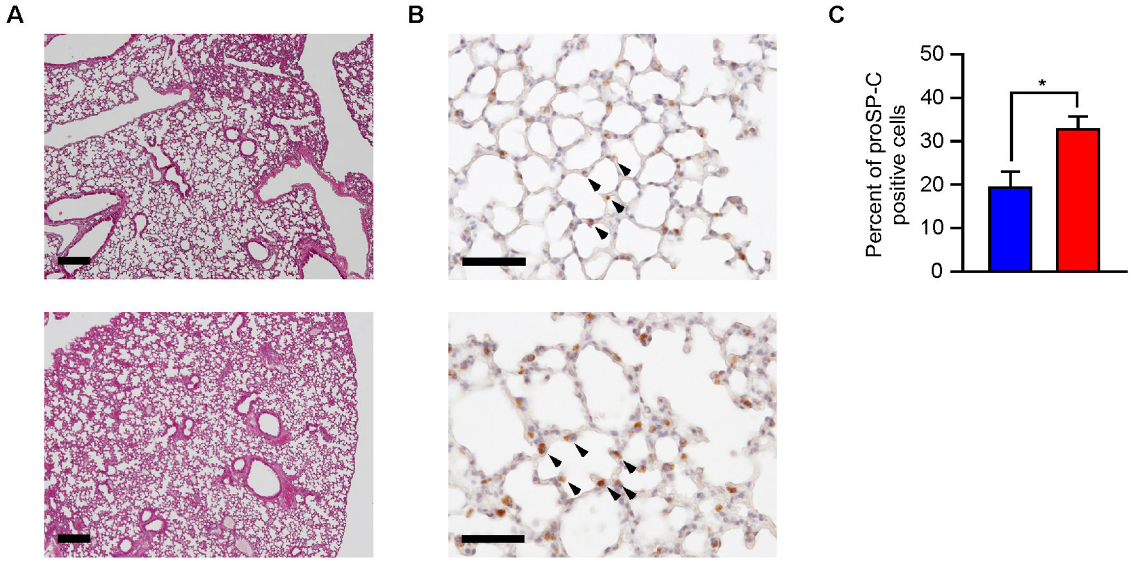

Figure S2 Increased number of Type II pneumocyte by the administration of LFK.

(**A**) Histology of lung tissue section stained with HE at DPI-0 (top: saline-administered mice, bottom: LFK-administered mice). Original magnification is X4. Scale bars indicate 250 μm. (**B**) Immunohistochemical staining of lung tissue section at DPI-0 using prosurfactant protein C (proSP-C) antibody (top: saline-administered mice, bottom: LFK-administered mice). Arrowheads indicate examples of proSP-C-positive cells. Original magnification is X40. Scale bars indicate 50 μm. (**C**) The proportion of proSP-C positive cells was counted in 6 random microscopic fields for each group at a magnification of X40 (blue: saline, red: LFK; *: $P < 0.01$, Student's *t* test).
