# Supplementary material for: Enterococcus faecalis FK-23 affects alveolar-capillary permeability to attenuate leukocyte influx in lung after influenza virus infection
Source: Springerplus. 2013 Jun 20;2:269. doi: 10.1186/2193-1801-2-269 (PMC3698428; doi:10.1186/2193-1801-2-269)
Supplement: Supplementary file 3 — Additional file 3: Figure S3: Cytokine and chemokine mRNA expression levels during the course of the viral infection. (A) Cytokine mRNA expression level in lung. (B) Chemokine mRNA expression level in lung. Each value is expressed as fold change compared to non-infected control mice (*: P < 0.01, Student’s t test). Black, blue, and red columns indicate non-infected control (n = 3), saline-administered (n = 6), and LFK-administered (n = 6) groups, respectively. (PDF 85 KB) [file 40064_2013_347_MOESM3_ESM.pdf]

Additional file 3: Figure S3

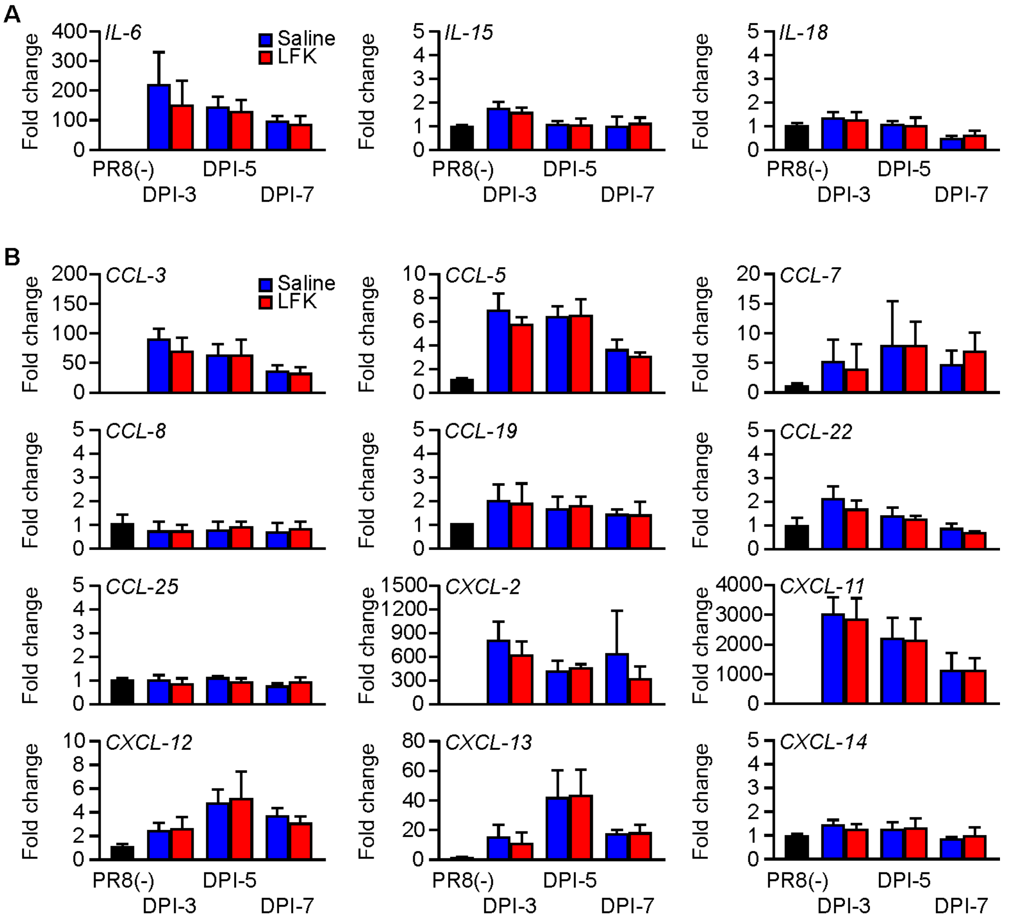

Figure S3 Cytokine and chemokine mRNA expression levels during the course of the viral infection.

(**A**) Cytokine mRNA expression level in lung. (**B**) Chemokine mRNA expression level in lung. Each value is expressed as fold change compared to non-infected control mice (*: $P < 0.01$, Student's *t* test). Black, blue, and red columns indicate non-infected control ($n = 3$), saline-administered ($n = 6$), and LFK-administered ($n = 6$) groups, respectively.
